# The presence of fever in adults with influenza and other viral respiratory infections

**DOI:** 10.1017/S0950268816002181

**Published:** 2016-10-03

**Authors:** A. A. CHUGHTAI, Q. WANG, T. C. DUNG, C. R. MACINTYRE

**Affiliations:** 1 School of Public Health and Community Medicine, UNSW Medicine, University of New South Wales, Sydney, Australia; 2 The Beijing Centre for Disease Prevention and Control, Beijing, China; 3 National Institute of Hygiene and Epidemiology (NIHE), Vietnam; 4 College of Public Service & Community Solutions, Arizona State University, Phoenix, AZ, USA

**Keywords:** Fever, influenza-like illness, viral infection

## Abstract

We compared the rates of fever in adult subjects with laboratory-confirmed influenza and other respiratory viruses and examined the factors that predict fever in adults. Symptom data on 158 healthcare workers (HCWs) with a laboratory-confirmed respiratory virus infection were collected using standardized data collection forms from three separate studies. Overall, the rate of fever in confirmed viral respiratory infections in adult HCWs was 23·4% (37/158). Rates varied by virus: human rhinovirus (25·3%, 19/75), influenza A virus (30%, 3/10), coronavirus (28·6%, 2/7), human metapneumovirus (28·6%, 2/7), respiratory syncytial virus (14·3%, 4/28) and parainfluenza virus (8·3%, 1/12). Smoking [relative risk (RR) 4·65, 95% confidence interval (CI) 1·33–16·25] and co-infection with two or more viruses (RR 4·19, 95% CI 1·21–14·52) were significant predictors of fever. Fever is less common in adults with confirmed viral respiratory infections, including influenza, than described in children. More than 75% of adults with a viral respiratory infection do not have fever, which is an important finding for clinical triage of adult patients with respiratory infections. The accepted definition of ‘influenza-like illness’ includes fever and may be insensitive for surveillance when high case-finding is required. A more sensitive case definition could be used to identify adult cases, particularly in event of an emerging viral infection.

## INTRODUCTION

Respiratory infections are common and one of the leading causes of morbidity and mortality, particularly in the extremes of age [1–3]. Influenza A and B, human rhinoviruses (HRV), respiratory syncytial virus (RSV), adenoviruses (ADV) and parainfluenza virus (PIV) are common respiratory viruses in adults and children [1–5]. Of respiratory infections, influenza is the most well studied viral infection, and is commonly reported (around 50%) as the cause of epidemics of respiratory infection, including nosocomial outbreaks [6]. Influenza virus is commonly isolated from febrile paediatric and elderly patients presenting with influenza-like illness (ILI) and acute respiratory illness (ARI) symptoms [1]. The accepted clinical case definition of ILI includes fever, which may be suitable for identifying paediatric cases, but less so for adults.

Fever is thought of as the most common presenting symptom of influenza in hospital emergency departments; however, the presence of fever depends on the age of person and the type of virus [7–10]. It is known that fever is less common in adults than children with influenza, and that adults may have atypical presentations [5, 6, 11]. In a matched case-control study in Finland, 317 laboratory-confirmed influenza cases and 353 controls with respiratory symptoms were recruited in children aged ⩽13 years. Fever was present in 89·8% (317/353) and 35·7% (126/353) of cases and controls, respectively [12]. In contrast to this, fever is not a common presentation in adults with laboratory-confirmed influenza. Monto *et al.* [13] examined clinical trial data of 3744 adult ILI cases (defined as body temperature ⩾37·8 °C or patients subjective feeling of feverishness) and of those 2470 (66%) had laboratory-confirmed influenza. Fever (⩾37·8 °C) was reported in 68% of laboratory-confirmed influenza cases, compared to 40% other ILI cases [13]. During a randomized clinical trial (RCT) around the efficacy of facemask and hand hygiene in the household setting, 44% (15/34) of secondary cases with influenza A and 32% (8/25) of cases with influenza B had fever history [14]. The rate of fever was 66% (137/207) in hospitalized influenza cases in a US study [15]. Another US study showed that less than half (42·4%) of healthcare workers (HCWs) with laboratory-confirmed influenza presented with fever [5]. Fever is a less common presenting symptom in elderly people which may lead to diagnostic and treatment delays [16]. In patients admitted with myocardial infarction, 9% had unrecognized and undiagnosed influenza on testing at admission, highlighting the low level of clinical suspicion of influenza [17].

The rate of fever also varies between influenza strains, being more common in influenza A strains than B, and higher in H3N2 [7–10, 18]. Fever is a commonly reported symptom during influenza outbreaks and pandemics due to novel and more virulent nature of strains. In China 67·4% of the patients infected by influenza A(H1N1)pdm09 had fever [19]. In another study in Beijing 465 suspected ILI cases were tested and of those 318 (68%) were positive for influenza virus (pandemic H1N1-165 and seasonal influenza H3N2-153) and all had history of fever [20].

The aim of this study was to compare the rates of fever in adult subjects with confirmed influenza and other respiratory virus infections and examine predictors of fever.

## METHODS

We analysed a dataset of laboratory-confirmed viral respiratory infections collected from three clinical trials of HCWs where active surveillance for respiratory viral illness was conducted in prospective follow up [21–23]. The same methods, data collection forms and outcome measures, were used across the three studies, allowing the data to be pooled [21–23]. Two studies were conducted in Beijing China: trial 1 (2008/2009) and trial 2 (2009/2010) and another study (trial 3) was conducted in Hanoi, Vietnam in 2010/2011 [21–23]. In all clinical trials, participants were asked to complete diary cards on a daily basis to collect information on number of working hours, patients seen, mask use hours, high-risk procedures performed and appearance of respiratory symptoms. Thermometers were given the participants to measure their temperature daily and at symptom onset. Symptomatic cases were asked to complete sick patient follow-up forms and detailed information was collected on the following symptoms: chill or fever, cough, congestion, runny nose, sore throat, sneezes, lethargy, loss of appetite, abdominal pain, muscle or joint aches. Swabs of both tonsils and the posterior pharyngeal wall were collected on the day of reporting.

In all RCTs, fever was defined as having body temperature ⩾38 °C. Clinical respiratory illness (CRI) and ILI were in the primary outcomes in three clinical trials. CRI was defined as two or more respiratory symptoms or one respiratory symptom and a systemic symptom and ILI was defined as fever ⩾38 °C plus one respiratory symptom [21–23].

### Analysis

We analysed data from all subjects with a positive isolation of a respiratory virus by multiplex polymerase chain reaction (PCR). Descriptive analysis was conducted for rates of fever by virus type. A logistic regression analysis was used to determine the predictors of fever. A multivariable log binomial model was fitted, using a generalized linear model to estimate relative risk (RR). All variables were included in initial model. In the final model, we included only those variables that were significant (*P* < 0·25) in initial analysis. A backward elimination method was used to remove the variables that did not have any confounding effect, i.e. could not make meaningful change (±10%) in the RR of the comparison arm. Finally we estimated the rates of CRI and ILI in the laboratory-confirmed viral respiratory infections and laboratory-confirmed influenza infections. The data was analysed using SAS v. 9.4 (SAS Institute Inc., USA).

### Ethical approval

Ethical approval of two clinical trials (China trials 2008–2009 and 2009–2010) were obtained from the Institutional Review Board and Human Research Ethics Committee of the Beijing Center for Disease Prevention and Control. For the Vietnam trial ethical approval was obtained from National Institute for Hygiene and Epidemiology (NIHE) (approval no. 05 IRB) and the Human Research Ethics Committee of the University of New South Wales (UNSW), Australia (HREC approval no. 10306). All participants provided written informed consent prior to commencing the trials.

## RESULTS

The demographic characteristics of 158 cases with laboratory-confirmed viral infections are presented in Table 1. Ninety (57%) cases were from China and 68 (43%) were from Vietnam. The mean age of HCWs was 32·8 years and most participants were nurses (65%) and female (87%). Most cases were non-smokers (92%) and had not received influenza vaccine (86%). Viruses isolated included rhinovirus (*n* = 75, 47%), RSV (*n* = 28, 18%), influenza (*n* = 13, 8%), PIV (*n* = 12, 8%), human metapneumovirus (hMPV; *n* = 7, 4%), coronavirus (*n* = 7, 4%) and ADV (*n* = 1, 1%). More than one virus was isolated in 15 cases (9·5%), including nine cases with influenza co-infection. Fever was documented in 23·4% cases (37/158) with a positive laboratory viral diagnosis.

**Table 1.** Demographic characteristics of cases in three clinical trials

Variable	No.	Percent
Country		
China (trials 1 and 2)	90	57
Vietnam (trial 3)	68	43
Profession		
Doctors	56	35·4
Nurses	102	64·6
Age, years, mean (s.d.)		32·8 (±9·14)
Gender		
Male	21	13·3
Female	137	86·7
Smoking status		
Current/ex-smoker	12	7·6
No	146	92·4
Influenza vaccine		
Yes	22	13·9
No	136	86·1
Virus type		
Co-infection	15	9·5
Human rhinovirus	75	47·5
Respiratory syncytial virus	28	17·7
Influenza A and B viruses	13	8·2
Other*	27	17·1
Fever		
Yes	37	23·4
No	121	76·6

* Other includes: parainfluenza virus (*n* = 12), human metapneumovirus (*n* = 7), coronavirus (*n* = 7) and adenovirus (*n* = 1).

Table 2 details rates of fever (⩾38 °C) associated with individual viral respiratory infections. HRV was the most common infection and 25·3% (19/75) of these had a fever. In 28 cases of RSV, four (14·3%) had fever; 8·3% (1/12) of PIV and 30% (3/10) of influenza A cases had fever. Seven cases of coronavirus and hMPV each were confirmed and of those two (28·6%) had fever. When cases with influenza and a co-infection were included, 36·4% (8/22) had fever.

**Table 2.** Rate of fever in respiratory infections in the pooled dataset of three clinical trials

Viruses isolated	No. of cases	Case with fever	Rate of fever (%)
Adenovirus	1	0	0
Coronavirus	7	2	28·6
Influenza A virus	10	3	30·0
Influenza A virus/human rhinovirus	3	3	100
Influenza B virus	3	0	0
Influenza B virus/human rhinovirus	2	2	100
Influenza B virus/parainfluenza	2	0	0
Human rhinovirus	75	19	25·3
Parainfluenza virus	12	1	8·3
Parainfluenza virus /coronavirus	1	0	0
Parainfluenza/influenza A virus	1	0	0
Respiratory syncytial virus	28	4	14·3
Respiratory syncytial virus/influenza A virus	1	0	0
Respiratory syncytial virus/human rhinovirus	3	0	0
Respiratory syncytial virus/parainfluenza	1	1	100
Human metapneumovirus	7	2	28·6
Human metapneumovirus/human rhinovirus	1	0	0
Total	158	37	23·4
All influenza*	**22**	**8**	**36·4**

* Includes co-infection with other viruses.

In univariate analysis, country, gender and smoking were significant predictors of fever. Country and smoking remained significant predictors in multivariate analysis while gender became non-significant. Fever rate was significantly higher in HCWs in Vietnam compared to HCWs in China [RR 2·99, 95% confidence interval (CI) 1·24–7·20]. Smokers were around five times more likely to have fever compared to non-smokers (RR 4·65, 95% CI 1·33–16·25). Virus type was not associated with fever in univariate analysis; however, after adjusting for other variables, rates of fever were significantly higher in HCWs co-infected with more than one virus compared to all other viruses excluding influenza (RR 4·19, 95% CI 1·21–14·52) (Table 3).

**Table 3.** Predictors of fever in cases with viral respiratory infections

Variable	Cases with fever (*n*/*N*)	Rate (%)	Univariate analysis (RR, 95% CI)	Multivariate analysis (RR, 95% CI)
Country				
China (trials 1 and 2)	15/90	16·7	Ref.	Ref.
Vietnam (trial 3)	22/68	32·4	**2·39 (1·13–5·07)**	**2·99 (1·24–7·20)**
Profession				
Doctors	12/56	21·4	0·84 (0·38–1·84)	
Nurses	25/102	24·5	Ref.	
Age			0·96 (0·92–1·01)	
Gender				
Male	9/21	42·9	**2·92 (1·12–7·62)**	
Female	28/137	20·4	Ref.	
Smoking status				
Current/ex-smoker	7/12	58·3	**5·41 (1·60–18·26)**	**4·65 (1·33–16·25)**
No	30/146	20·5	Ref.	Ref.
Influenza vaccine				
Yes	6/22	27·3	1·27 (0·46–3·52)	
No	31/136	22·8	Ref.	
Type of virus				
Influenza virus	3/13	23·1	1·09 (0·28–4·24)	1·89 (0·43–8·31)
All other viruses*	28/130	21·5	Ref.	Ref.
Co-infection	6/15	40	2·43 (0·80–7·40)	**4·19 (1·21–14·52)**

RR, Relative risk; CI, confidence interval.

* Includes human rhinovirus (*n* = 75), respiratory syncytial virus (*n* = 28), parainfluenza virus (*n* = 12), human metapneumovirus (*n* = 7), coronavirus (*n* = 7) and adenovirus (*n* = 1).

CRI symptoms were present in 84·8% (137/158) of HCWs with laboratory-confirmed viral infections and 90·9% (20/22) laboratory-confirmed influenza infections. The corresponding rates of ILI in the two groups were 9·5% (15/158) and 13·6% (3/22), respectively.

## DISCUSSION

We have shown, using prospectively collected data, that the rate of fever in adults with confirmed viral respiratory infections is much lower than described in children [1, 9]. The standard clinical case definition of ILI requires fever to be present – the majority of influenza cases in this series would have been missed using the ILI definition. This has implications for effective triage, early antiviral treatment and preventive measures for adults with influenza, particularly during outbreaks and pandemic situations. For other respiratory infections, clinical case definitions need to be more sensitive, or >75% of cases will be missed. The main implication for future surveillance, measurements and research studies is that the ILI case definition in adults may be highly insensitive. For some types of surveillance systems, this may not be an issue, but for diagnostic screening in event of an emerging viral infection (such as for triage and implementation of infection control protocols) [24, 25], a more sensitive case definition is needed.

Rates of fever in influenza and other viral respiratory infections in this study were lower compared to other studies which report fever in around 50–70% adult cases [1, 5, 13, 15]. However, this variation may be due to different study base, case definition and viral strains, as well as the prospective measurement of incident infections. Many research studies use fever as an inclusion criterion for laboratory testing [13, 26]. While this may be suitable for studies in children, it is not adequately sensitive for studies of adults, as we have shown the majority of confirmed cases will be missed. The cut-off point for fever could be another factor in sensitivity. Some studies have set lower cut-off points for fever, and report higher rates of fever in laboratory-confirmed influenza cases [13, 27]. Carrat *et al*. collected data of cases presented in 35 general practices in France and collected nasal swabs from suspected influenza cases and defined fever as ⩾37·8 °C. They found fever in influenza A(H3N2), influenza A(H1N1) and influenza negative cases in 95·2%, 77·5% and 72·7%, respectively. Applying a cut-off of ⩾38·2 °C, the corresponding rates are 82·2%, 59·3% and 43·9% [27]. Symptoms of feverishness (subjective feeling of fever) are included in ILI definitions in some cases [13].

Previous studies report high rates of fever in children compared to the adults [11]. Low rates of fever in adults may also be due to protection via cross-reactive antibodies due to age-dependent differences in the immunity [7, 28]. Continued exposure to influenza throughout life may result in a broader protection with age. Infection may provoke a stronger immune response in children with minimal to no exposure history compared to adults. Therefore influenza infection history might help explain potential differences in clinical symptom severity (and presence of fever) between children and adults.

A recent study reported high rates of influenza and other respiratory virus in afebrile HCWs with only respiratory symptoms [5]. Of 22 laboratory-confirmed influenza cases in this study, only three (13·6%) had ILI symptoms, which is very low compared to other studies. In a prospective influenza surveillance study, ILI symptoms were present in 48% of adults and 61% of children with laboratory-confirmed influenza virus [1]. CRI symptoms were present in 90·9% (20/22) of laboratory-confirmed influenza cases in this study.

A highly sensitive definition of influenza may be required to diagnose most of adult influenza cases in the clinical setting to ensure rapid treatment and isolation, and prevention of nosocomial transmission. Inclusion of ILI cases may overestimate the proportion of febrile cases in influenza surveillance given fever is included in the definition. Pre-symptomatic and asymptomatic influenza cases will also be missed, although infectivity and transmissibility of these cases is yet to be proven [29]. Longitudinal studies, where all participants are tested, provide similar estimates around rates of fever as in our study [14]. We propose a more sensitive clinical case definition without fever as a requisite criterion.

Clinical signs and symptoms are less studied for other viral respiratory infections, but available evidence suggests that other respiratory viruses are associated with a lower rate of fever compared to influenza [5, 30–33]. Putto and colleagues [30] examined the clinical records of 258 children (>3 months) in a large hospital in Finland, including ADV (25 cases), influenza A and B (74 cases), PIV (99 cases) and RSV (60 cases). Fever (⩾39·0 °C) was recorded in 68% cases with ADV, 84% influenza A virus, 65% influenza B, 41% PIV-1, 50% PIV-2, 47% PIV-3, and 52% RSV. Van den Hoogen and colleagues estimated the prevalence and clinical symptoms of hMPV infection, in The Netherlands and fever was reported in 61% of the hMPV-positive cases [31]. In Hong Kong, hMPV was found in 5·5% (32/587) of children admitted in hospitals and all had fever [32]. Manoha *et al*. examined nasal wash specimens from 931 hospitalized children and found hMPV (6%), RSV (28·5%), rhinoviruses (18·3%), influenza A (6%), PIV-1 (0·2%) and PIV-3 (0·3%). Fever was reported in 39·2% cases with hMPV, 37·8% cases with RSV and 30·2% with rhinovirus [33]. Of the 210 elderly patients with influenza and 145 with RSV, fever was reported in 65% and 50%, respectively [3]. A US study also reported low rates of fever in HCWs infected with coronavirus 229E (13·5%), coronavirus HKU (11·4%), coronavirus NL63 (31·3%) and RSV (12·9%) and all cases of hMPV were without fever [5]. Rate of fever for all other viruses (excluding influenza) was 21·5% (28/130) in this study.

Co-infection with more than one virus was the strongest predictor of fever for adults with confirmed viral respiratory infections in the present study. Previous studies also show high rates of fever in cases with dual respiratory viral infections compared to single viral infection [34, 35]. Rates of hospitalization and ICU admission are also reported to be higher in cases with dual respiratory viral infections [36–38]. Increased severity of symptoms in co-infection cases might be due to an altered immune response [34]. Around 10% (15/158) of cases in our dataset were infected with more than one virus. Drews *et al*. reviewed the data of eight prospective epidemiological studies and reported the rate of co-infection was 5% [37]. Studies in children generally report higher rates of co-infection cases (17–20%) [34, 35, 38, 39]. Clinicians should consider the possibly of co-infection if a patient presents with fever; however, further epidemiological and clinical studies are required.

Smoking was also a significant predictor of fever in this study. Smoking increases the risk of viral and bacterial infections through changes in respiratory epithelial and altered immune response [40–43].The risk of influenza also increases several times in smokers, compared to non-smokers [40]. Atypical clinical presentation of influenza and other respiratory infections in adults could be due to altered structural and immune response associated with active/passive smoking and other environmental hazards. The mechanism by which smoking increases the risk of fever is not clear. High rates of fever in smokers may also be due changes in immunoglobulin levels which could increase viral load. The severity of symptoms generally increases when high viral load is detected in the blood [44].

The difference in fever rates between China and Vietnam may be due to prevalence of viruses and co-infection. RSV was the most commonly isolated pathogen from China (31%), followed by rhinovirus (20%) and influenza virus (13%). In contrast to this HRV was the most commonly isolated pathogen from Vietnam (85·3%). The number of cases with co-infection were also different in the two countries – 13 (14%) in China and two (3%) in Vietnam. In multivariate analysis, we adjusted for country and type of virus. Limited data are available regarding the prevalent viruses circulating in China during the study period. For the trial 1 period, all influenza was influenza A(H1N1)pdm. For the trial 2 period, 21·3% were H1N1pdm, 2·9% were H3N2, 3·0% were influenza B Victoria, 2·6% were influenza B Yamagata, 71·2% were influenza A unsubtyped (Y. Zhang, Beijing Centre for Disease Prevention and Control, personal communication). We could not obtain data on the viruses circulating in Vietnam during the study period.

There are some limitations to this study. We did not subtype the influenza strains, and studies show that the rate of fever also varies between influenza strains [7–10, 18]. Fever data was self-reported but self-measured in three trials using a traditional glass and mercury thermometer. Lower fever rates in Chinese HCWs in this study might be due to due to differences in circulating viruses (and their pyrogenicity) between the two countries when the studies were conducted. A Japanese study of children with influenza reported a tendency towards shorter duration of fever with increasing age in children [18]; however, age and other demographic characteristics were not significant in that study.

## CONCLUSION

Compared to children, this study shows that adults are less likely to have fever with a respiratory viral infection, even influenza. The implication of this finding is that for rapid treatment and reducing the risk of transmission of infection, clinicians should be aware that a diagnosis of viral respiratory infection, even influenza, is possible in the absence of fever. Many of these infections are transmissible even when infected persons are asymptomatic or pre-symptomatic, and greater vigilance for respiratory symptoms in HCWs could reduce nosocomial transmission of respiratory viral infections. The absence of fever should not preclude a differential diagnosis of influenza or other respiratory viruses in adults.
